# Manifestations and Impacts of Racial Aggressions on Black Individuals’ Health and Well-Being in University Settings Across Organization for Economic Cooperation and Development Countries: Protocol for an Integrative Review

**DOI:** 10.2196/92748

**Published:** 2026-09-25

**Authors:** Gisèle Mandiangu Ntanda, Judith Lapierre, Naima Bouabdillah, Veronica Livia Tavalica, Geneviève Rouleau, Ruth Ndjaboue, Drissa Sia, Natalie Stake-Doucet, Catherine Seguin, Gina Lafortune, Leonel Philibert

**Affiliations:** 1 Department of Nursing, Université du Québec en Outaouais, 5, rue Saint-Joseph, Saint Jérôme, QC, J7Z 0B7, Canada, 1 4182643393; 2 Faculty of Nursing, Université Laval, Québec, QC, Canada; 3 Department of Nursing, Université du Québec à Trois-Rivières, Trois Rivières, QC, Canada; 4 Department of Nursing, Université du Québec en Outaouais, Gatineau, QC, Canada; 5 School of Social Work, Université de Sherbrooke, Sherbrooke, QC, Canada; 6 Department of Special Education and Training, Université du Québec à Montréal, Montréal, QC, Canada; 7 Library Services, Université du Québec en Outaouais (UQO), Saint-Jérôme, QC, Canada; 8 Human Diversity Hub, Université de l’Ontario Français, Toronto, ON, Canada

**Keywords:** aggression, minority health, perceived discrimination, social discrimination, racial minorities, minority groups, Black individuals

## Abstract

**Background:**

The number of international migrants continues to rise globally, particularly in countries belonging to the Organization for Economic Cooperation and Development (OECD). This growing diversity presents sociopolitical challenges related to social cohesion, which are also reflected in academic settings. Some individuals are exposed to different forms of discrimination based on skin color, including both racial microaggressions and macroaggressions. Racism is a major determinant of health and has deleterious effects on those exposed to it.

**Objective:**

This protocol aims to analyze and synthesize existing knowledge on racial aggressions, both macroaggressions and microaggressions, experienced by Black individuals as students or members of the teaching staff (including lecturers and professors) in university contexts across OECD countries. The research question posed for this purpose is as follows: What does the scientific literature reveal about racial aggressions (both macroaggressions and microaggressions) experienced by Black individuals as students or members of the teaching staff in university contexts across OECD countries?

**Methods:**

This integrative review protocol is developed in accordance with the PRISMA-P (Preferred Reporting Items for Systematic Reviews and Meta-Analyses Protocols) guidelines. An integrative review will be conducted following the framework of Whittemore and Knafl. It will be carried out in 7 distinct phases: (1) problem identification, (2) literature search, (3) study selection, (4) data evaluation, (5) data extraction and analysis, (6) data synthesis, and (7) presentation of findings. A literature search will be conducted using electronic databases such as CINAHL, PubMed/MEDLINE, PsycINFO, Scopus/Web of Science, and Embase to identify studies addressing various forms of racial aggression in academic environments. The methodological framework of Whittemore and Knafl will guide the entire review process, from study selection to data analysis and synthesis. The quality assessment of the studies included in this integrative review will be conducted using the Mixed Methods Appraisal Tool (MMAT; 2018 version).

**Results:**

The study identification and selection process began in September 2025. A total of 7098 records were identified through electronic database searches. The literature to be reviewed is expected to include studies conducted across multiple OECD countries involving diverse university populations, such as students, faculty, and administrative staff, and using a range of methodological approaches. This integrative review will examine how racial aggressions (both microaggressions and macroaggressions) manifest in academic settings, the strategies developed by Black individuals to cope with these experiences, and their impacts on health and well-being. Study completion is anticipated between August and September 2026.

**Conclusions:**

The findings will inform future research on racial aggressions in higher education and contribute to the development of evidence-based prevention strategies. Ultimately, the review aims to support the development of university policies and programs that promote inclusive environments and ensure equitable access to resources and opportunities for all students and staff.

## Introduction

### Background

The global number of international migrants continues to rise, reaching more than 280 million in 2020 [1]. This increase reflects a significant shift in global mobility, as individuals relocate for diverse reasons, including escaping repression, insecurity, and poverty. According to the Organization for Economic Cooperation and Development (OECD) [2], the 5 countries that received the highest numbers of new asylum applications in 2023 were the United States (1,176,050), Germany (329,120), Spain (160,470), Canada (146,730), and France (145,210). For instance, Canada ranked fourth among OECD countries, marking a 55% increase compared with 2022, when it ranked eighth. In 2021, Canada welcomed more than 8.3 million migrants, representing nearly one-quarter (23.0%) of its total population [3], positioning it among the most multicultural nations globally.

Among new arrivals to OECD countries, an increasing number are migrating for educational purposes. Universities in these countries attract large cohorts of international students annually from diverse global regions. This trend, known as the *internationalization of higher education*, continues to grow [4]. In Canada, for instance, 682,889 study permit holders entered the country in 2023 [5], reflecting significant diversity in terms of race, culture, religion, and social background.

This diversity gives rise to sociopolitical challenges related to coexistence, including the interaction of different cultures, identity tensions, and unequal access to resources and opportunities within academic environments. It also places institutional capacities under strain, particularly in terms of their ability to embrace, recognize, and value otherness [6-9]. This is reflected, for instance, in efforts to promote the academic success of students from racialized minority groups, implement inclusive and antiracist policies, and integrate non-Western knowledge and lived experiences into curricula.

However, within this social context, where multiple realities and diverse identities intersect, many racialized individuals encounter a range of personal and systemic barriers in their daily lives as they seek acceptance, recognition, and validation [6,9,10]. In academic settings in particular, individuals from visible-minority groups are disproportionately affected by discrimination [10,11]. Such discrimination may manifest in the form of racial microaggressions and macroaggressions (RMMAs), biased academic evaluations, social exclusion, and limited access to mentoring and professional development opportunities. RMMAs, although often subtle and invisible, constitute very real forms of violence that individuals may experience due to their skin color, accent, or religious affiliation [11,12].

Racial macroaggressions refer to systemic and institutionalized forms of prejudice and oppression. These can manifest, for example, within the education system, the workplace, the health care system, the legal and policing systems, and more. Systemic racism is a form of macroaggressions that encompasses a whole system of policies, laws, practices, attitudes, and societal structures that produce discrimination based on skin color or national origin [11].

Racial microaggressions, on the other hand, refer to incivilities, verbal assaults, hostile gestures, disrespectful comments, or negative attitudes directed at an individual or group based on phenotypic or cultural characteristics. These often occur unconsciously, not only through subtle and disdainful looks, gestures, or tones but also through indifference, deliberate ignorance, or systematic exclusion from social interactions. They are pervasive in everyday conversations and interactions and are often perceived as harmless or innocent [11,12].

A growing body of evidence indicates that Black individuals are disproportionately exposed to racism in academic settings, including overt discrimination and everyday microaggressions [13-17]. In nursing education, qualitative studies have shown that Black students frequently experience microaggressions from peers and faculty, as well as in clinical environments, which can negatively affect their learning and contribute to psychological distress [16]. Moreover, these experiences occur within broader structural inequities, including underrepresentation and systemic barriers that hinder academic success and a sense of belonging among Black nursing students [15].

RMMAs have a significant impact on the well-being, academic performance, and sense of belonging of those affected [18-22]. Exposure to racism is associated with significant adverse health outcomes, including increased symptoms of anxiety, depression, and sleep disturbances, particularly in the context of more overt discrimination [23-25]. It is also linked to physiological stress responses, such as elevated blood pressure, as well as maladaptive coping behaviors, including alcohol misuse and other health-risk behaviors [26-28].

Despite institutional commitments to equity, diversity, and inclusion, academic environments in OECD member countries continue to be marked by racial aggressions targeting Black individuals [29-31]. These acts of aggression, often minimized or overlooked, take various forms, including microaggressions, systemic discrimination, and symbolic exclusion. Such discriminatory practices restrict access to employment opportunities, support services, and funding for research and scholarships. This unequal access to resources and opportunities directly contributes to the persistence and reinforcement of both social and health inequalities [21,32].

The various forms of RMMAs at universities in OECD countries have not yet been extensively studied [33]. Addressing RMMA remains a “taboo” subject in several countries. For example, in northern European countries, such as Sweden and Norway, although racism is recognized as a problem, it is very difficult to address it publicly without provoking resistance or without the discussion quickly turning into a debate on freedom of expression, which can minimize or deflect criticism [34]. As a result, it is difficult to develop and implement effective strategies that would better prevent these aggressions. It is important to note that institutional policies vary greatly from one country to another, from one administrative region to another, and even from one academic institution to another. These policies are constantly evolving and require research support to document, evaluate, and renew them.

In this context, an integrative review is needed to synthesize the existing knowledge, identify gaps, and inform practices and policies that can effectively prevent and respond to racial aggression in higher education institutions. More specifically, we chose to focus on Black individuals, as they experience distinct forms of discrimination, often linked to anti-Black racism, which shape their experiences within academic environments [10,11,20]. Anti-Black racism is rooted in a historical legacy shaped by colonialism and slavery [35,36], which has contributed to the construction and persistence of racial hierarchies that continue to influence contemporary institutions.

Documenting the racism experienced by Black individuals is essential to make visible systemic inequities, understand their impact on health and well-being, and inform the development of contextually relevant and effective antiracism interventions. Despite growing evidence of the impact of racial discrimination on the health and well-being of Black individuals [20,22], the literature remains fragmented, particularly regarding the forms of racial aggressions and the coping strategies developed to address them in academic settings. Moreover, there is a lack of an integrative synthesis that comprehensively examines how these forms of racial aggressions manifest, how Black individuals respond to them, and the implications for health and overall well-being.

The integrative review proposed in this protocol aims specifically to address this gap by providing a comprehensive synthesis of the existing literature.

### Objectives and Research Question

This integrative review aims to analyze and synthesize existing knowledge of racial aggressions, both macroaggressions and microaggressions, in academic settings, as experienced by Black individuals as students or members of the teaching staff (including lecturers and professors) in university settings across OECD countries. In this regard, it will answer the following research questions:

1. How do racial aggressions (macroaggressions and microaggressions) manifest in academic settings across OECD countries?
2. What strategies do Black individuals develop to deal with racial aggressions in academic settings across OECD countries?
3. What strategies have academic institutions implemented to prevent, address, and respond to racial aggressions experienced by Black individuals in academic environments?
4. How do racial aggressions (macroaggressions and microaggressions) in academic settings across OECD countries affect the health and well-being of Black individuals?

By examining the manifestations of racial aggressions, the coping strategies developed by Black individuals, and the institutional responses implemented, this review seeks to provide a comprehensive and integrated understanding of how these experiences collectively influence health and well-being.

## Methods

### Design

This integrative review protocol is reported in accordance with PRISMA-P (Preferred Reporting Items for Systematic Reviews and Meta-Analyses Protocols) guidelines [37] (Checklist 1). The integrative review will be conducted following the methodological framework proposed by Whittemore and Knafl [38]. This approach allows for the inclusion of studies using diverse methodologies—quantitative, qualitative, and mixed methods—thus offering a comprehensive understanding of the phenomenon under investigation. The use of an integrative review is particularly well suited to our study, as it enables a multidimensional exploration of macro-level and micro-level racial aggression in academic settings. It also helps identify gaps in the existing literature, supports the development of new conceptual or theoretical frameworks, and informs university policies aimed at fostering more inclusive environments. The review will be conducted in 7 distinct phases: (1) problem identification, (2) literature search, (3) study selection, (4) data evaluation, (5) data extraction and analysis, (6) data synthesis, and (7) presentation of findings.

### Problem Identification

The identified problem is presented in the “Background” section of the protocol. The research process began with discussions among the research team to define the primary objective and formulate the research questions. Once these elements had been established, a working session was conducted with a professional librarian, who is also a coauthor of this study, to develop and validate the search strategies.

Following this, the research team received training in database searching and screening procedures. The identification of relevant studies was subsequently carried out through systematic searches of electronic databases using validated search strategies. The search process was designed to ensure comprehensive coverage of literature relevant to the research questions.

### Literature Search

#### Databases

An exhaustive literature search will be conducted across databases to extract relevant information on the topic and its dimensions. Five databases will be used to conduct the literature review—CINAHL, PubMed/MEDLINE, PsycINFO, Scopus/Web of Science, and Embase—to search for papers presenting various forms of racial aggressions observed in universities across OECD countries. These databases were selected to ensure comprehensive and interdisciplinary coverage of the topic by capturing literature from the fields of health, psychology, and the social sciences. Given the health-focused perspective of this integrative review, databases such as CINAHL, PubMed/MEDLINE, and Embase were prioritized for their strong coverage of health and clinical research. PsycINFO was included to capture psychological dimensions, particularly those related to coping strategies, whereas Scopus/Web of Science was selected to broaden the search across multidisciplinary and international literature.

In the context of globalization and rapid change accentuated by the emergence of new communication technologies, it is recommended that the literature search be limited to a recent period, ideally the last 10 years (2015‐2025). This approach ensures that the data and analyses consulted reflect current developments in the field, incorporate recent technological innovations, and consider recent socioeconomic and cultural changes that directly influence the phenomenon under study [39]. However, preliminary exploratory searches conducted for this review revealed a limited number of publications addressing racial aggressions in academic settings within on this issue in OECD countries. Therefore, applying such a temporal restriction could lead to the exclusion of relevant studies and hinder a comprehensive understanding of the topic. For this reason, no date limits will be applied in the initial search for this integrative review, allowing for a more inclusive and exhaustive synthesis of the available literature [33].

The database search process will be carried out with the support of the librarian at the Université du Québec en Outaouais (UQO). Two members of the research team will simultaneously and independently verify the results obtained from the search equations to ensure the reliability and consistency of the results. Once these results have been verified, they will be imported into Rayyan, a software application dedicated to paper selection, to facilitate the sorting and evaluation of relevant studies according to the review criteria.

#### Inclusion and Exclusion Criteria

This integrative review will include studies that meet the following criteria: (1) participants are adults (aged 18 y or older), Black individuals living in OECD countries who are engaged in academic environments, either as students or members of the teaching staff (including lecturers and professors); (2) studies that focus on the manifestations of racial aggressions, both macroaggressions and microaggressions, in university settings across OECD countries; (3) studies that examine the impacts of racial aggressions on the physical health and psychological well-being of Black individuals; (4) studies that explore coping and resistance strategies used by Black individuals to navigate and respond to such experiences in academic environments; (5) studies that analyze the strategies implemented by academic institutions to prevent, address, and respond to racial aggressions experienced by Black individuals; and (6) studies that are published in English or French.

For the purposes of this review, “Black individuals” refers to people of African descent or those who identify as Black based on cultural, historical, and racial identity. This includes members of racialized communities such as African Americans, Afro-Caribbeans, and people of African heritage living in various regions worldwide.

Although we acknowledge that institutional policies and contexts vary across countries and educational institutions, the inclusion of OECD countries was guided by the need to examine racial aggressions within relatively comparable higher education systems. OECD countries share certain structural and policy characteristics, which facilitate the identification of common patterns and variations in the manifestations of racial aggressions, coping strategies, and health impacts across different academic contexts.

Studies were excluded if they met any of the following criteria: (1) conducted outside academic environments (eg, workplaces or community settings), (2) focused exclusively on other forms of discrimination (eg, gender or disability) without addressing racial dimensions, or (3) classified as editorials, commentaries, case studies, government reports, conference papers, study protocols, trial registrations, unpublished theses or dissertations, or other forms of gray literature.

#### Search Terms and Types of Searched Literature

The main keywords will be racial aggressions/racism, academic/university settings, Black individuals, and OECD countries (Multimedia Appendix 1). Relevant synonyms will be used to facilitate the identification of relevant studies. A meeting was held with the UQO librarian to validate the search strategy (Table 1).

**Table 1.** Search strategy.

Number	Queries in CINAHL
1	MH (“racism” OR “racist” OR “racial bias” OR “racial prejudice” OR “racial discrimination” OR “ethnic discrimination” OR “cultural bias” OR “anti-black” OR “systemic racism” OR “structural racism” OR “racial inequity” OR “racial inequality” OR “racialized” OR “microaggression*” OR “race-based”)
2	MH (universit* OR université* OR universidad* OR universita* OR universität* OR universidade* OR college* OR campus* OR “higher education” OR “postsecondary education” OR “tertiary education” OR “vocational school” OR polytechnic OR institute* OR “technical college” OR “faculty”)
3	MH (“racialized people” OR “racialized person*” OR “racialized group*” OR “racialized communit*” OR “racial minorities” OR “ethnic minorities” OR “minority group*” OR “minority population*” OR “people of color” OR “persons of color” OR “communities of color” OR “people of color” OR “persons of color” OR “communities of color” OR “visible minority” OR “visible minorities” OR “equity-deserving group*” OR “equity-seeking group*” OR “underrepresented group*” OR “underrepresented minority” OR “marginalized population*” OR “marginalized communit*” OR “racial and ethnic group*” OR “ethnocultural group*” OR “Black people” OR “Black individuals” OR “African descent” OR “African American” OR “Afro-descendant*” OR “BIPOC” OR “IBPOC” OR “POC” OR “population racisée” OR “minorité visible” OR “groupes racisés”)
4	MH (“OECD countries” OR “Australia” OR “Austria” OR “Belgium” OR “Canada” OR “Chile” OR “Colombia” OR “Costa Rica” OR “Czechia” OR “Czech Republic” OR “Denmark” OR “Estonia” OR “Germany” OR “Finland” OR “France” OR “Greece” OR “Hungary” OR “Iceland” OR “Ireland” OR “Israel” OR “Italy” OR “Japan” OR “Latvia” OR “Lithuania” OR “Luxembourg” OR “Mexico” OR “Netherlands” OR “New Zealand” OR “Norway” OR “Poland” OR “Portugal” OR “Slovenia” OR “Slovakia” OR “South Korea” OR “Republic of Korea” OR “Spain” OR “Sweden” OR “Switzerland” OR “Turkey” OR “UK” OR “USA” OR “United States”)

The search strategy for each database, adapted to its specific context, is presented in Multimedia Appendix 2.

### Study Selection

Two reviewers will independently select the literature to be included in the review, following a 2-step selection process using the Rayyan platform and in accordance with the predefined inclusion and exclusion criteria. The first step after removing duplicates will be to screen titles and abstracts to identify relevant documents based on predefined eligibility criteria. Studies meeting the initial screening criteria will be retained for full-text review. In the second step, the full texts of the selected papers will be assessed to confirm eligibility.

All reasons for exclusion will be systematically documented. In cases of disagreement among the 3 evaluators, a discussion will be held to reach a consensus. If consensus cannot be achieved, the third evaluator will serve as an arbitrator to make the final decision. The entire paper selection process will be transparently summarized using a PRISMA-P flow diagram.

### Data Evaluation

Due to the integrative nature of this review, a standard risk-of-bias assessment will not be conducted. The quality assessment of the studies included in this integrative review will be conducted using the Mixed Methods Appraisal Tool (MMAT; 2018 version) [40], which is recognized for its relevance in evaluating qualitative, quantitative, and mixed methods research. This step will be carried out rigorously, in collaboration with researchers who possess specific methodological expertise: quantitative method specialists for the appraisal of quantitative studies and qualitative method specialists for the appraisal of qualitative studies. This collaborative approach aims to ensure the validity and reliability of the critical analysis of the selected sources.

### Data Extraction and Analysis

After selecting the relevant studies, a systematic data extraction and mapping strategy will be implemented to organize the information according to the type of racial aggression (“micro” or “macro”) that occurred in universities across various OECD countries. An extraction grid (Multimedia Appendix 3) will be developed with the research team to systematically collect relevant information from the selected studies. The software for data extraction and presentation will be Microsoft Excel and Rayyan.

Three team members will participate in the data extraction process. Data analysis will be conducted in 2 stages. First, the studies will be categorized based on the type of racial aggression identified in the selected papers, either microaggressions or macroaggressions. Subsequently, a thematic synthesis will be conducted to integrate the findings related to coping strategies and health outcomes.

We will use a concept-mapping approach, defined by Osei-Kwasi et al [41], as “seeing things as a whole,” inspired by system thinking. This method involves several steps: generating a list of factors, classifying them, and then structuring them into groups by consensus based on their interrelationships [41]. This grouping will help develop a systemic framework illustrating the manifestations of racial aggressions experienced by Black individuals in academic settings in developed countries, and how the intersection of these manifestations affects their well-being.

### Data Synthesis

A qualitative, integrative approach will be used to synthesize the findings across studies. Results will be organized around the main categories of racial aggression (microaggressions and macroaggressions) and presented within key thematic domains that reflect their manifestations, contexts, and affected populations.

A synthesis table will be developed to present the key characteristics of the included studies, including authorship, year of publication, country, research objectives, study design, population characteristics, reported findings, and study limitations. This table will support structured comparison across studies. It will be performed using both tabular and narrative approaches. The narrative synthesis will be structured around predefined analytical categories aligned with the objectives of the review.

The synthesis will highlight the relationships among different forms of racial aggression and incorporate an intersectional perspective to capture the complexity of these experiences. The findings will also be structured according to their impact on well-being (psychological, academic or professional, and social). The results will be presented in a summary table to map the studies according to the identified categories and subcategories. The results obtained will highlight the main trends as well as gaps in the literature that require special attention in future research.

Potential meta-biases, including publication bias, selective reporting, and language bias, will be considered during the interpretation of the findings. Efforts will be made to include studies from diverse sources and contexts to minimize the impact of these biases. However, due to the heterogeneity of study designs and the nature of the integrative review, a formal statistical assessment of meta-biases will not be performed.

### Presentation of Findings

As part of an integrative review, data presentation is a critical step that goes beyond merely listing the findings. Its purpose is to organize, compare, and interrelate the collected information to highlight both the specific contribution of each study and the overarching understanding that emerges from the entire body of work.

To this end, a synthesis table will be developed. It will provide a clear and structured overview of the included studies, listing key elements for each study, such as the authors and year of publication, the country where the research was conducted, the research objectives, the methodology used, the characteristics of the study population, the main findings, and the identified limitations. This format will facilitate comparison across studies and highlight areas of convergence and divergence.

As a complement, a narrative presentation will be developed in the form of an in-depth textual synthesis, structured around predefined analytical categories. These may include, for example, contextual factors, lived experiences, and proposed interventions. Each section will be illustrated with findings from the selected studies, thereby enhancing the understanding of the phenomenon under investigation and offering an integrated analysis that goes beyond the mere juxtaposition of individual data.

## Results

The integrative review outlined in this protocol is part of a research project funded by the Social Sciences and Humanities Research Council through the 2025 Insight Grant competition. The study identification and selection process began in September 2025. A total of 7098 records were identified through electronic database searches. Following the removal of duplicates, records were screened based on titles and abstracts in accordance with predefined inclusion and exclusion criteria. Studies deemed potentially relevant were subsequently assessed for eligibility through full-text review. Eligible studies were then subjected to a methodological quality appraisal to ensure scientific rigor. Following this rigorous selection process, 52 studies were included in the review.

The review is currently in the data synthesis phase, and completion is anticipated between August and September 2026. The retrieved literature is expected to include studies conducted across multiple OECD countries, involving diverse university populations such as students, faculty, and administrative staff, using a range of methodological approaches (quantitative, qualitative, and mixed methods). Any modifications to the original protocol, including changes to inclusion criteria or search strategies, will be documented and justified to ensure transparency.

## Discussion

The integrative review outlined in this protocol aims to analyze and synthesize existing knowledge of racial aggressions, both macroaggressions and microaggressions, experienced by Black individuals as students or members of the teaching staff (including lecturers and professors), in university contexts across OECD countries. Across the literature, racism is most often described as an everyday, normalized, and structurally embedded phenomenon rather than an overt or exceptional one. Students and faculty experience racism through microaggressions, stereotyping, exclusion from learning opportunities, biased assessment, and silencing when racism is reported [31,42,43].

Racism is a significant social determinant of health [21,44]. By examining the manifestations of racial aggressions, the coping strategies developed by Black individuals, and the institutional responses that have been implemented, this review seeks to provide a comprehensive and integrated understanding of how these experiences collectively influence health and well-being.

By synthesizing empirical and theoretical literature, this review aims to provide a comprehensive understanding of both macro and micro racial aggression in academic settings across OECD countries (Multimedia Appendix 1). Examining both macro-level and micro-level dynamics highlights how systemic inequities and everyday interactions intersect within broader historical and institutional contexts, including systemic racism and colonial legacies.

This review also seeks to synthesize the coping strategies developed by Black individuals, addressing the lack of integrative work that captures their diversity and contextual influences. Adopting a health-focused perspective, it positions racial discrimination as a key social determinant of health. The findings are expected to contribute to the literature by addressing existing gaps, informing future research, including longitudinal and intersectional approaches, and guiding the development of targeted, culturally relevant interventions and policies that promote equity in academic settings.

A key strength of this integrative review lies in its capacity to incorporate diverse study designs, enabling a holistic understanding of the complex and multidimensional phenomena. However, limitations include the potential risk of subjective interpretation and variability in terminology across disciplines. To mitigate these challenges, the review will involve multiple independent reviewers, standardized appraisal tools, a transparent audit trail, and a comprehensive search strategy incorporating controlled vocabulary and diverse keywords.

## Supplementary material

10.2196/92748 Multimedia Appendix 1 Organization for Economic Cooperation and Development countries and capitals.

10.2196/92748 Multimedia Appendix 2 Detailed search strategies for other databases.

10.2196/92748 Multimedia Appendix 3 Data extraction tool.

10.2196/92748 Checklist 1 PRISMA-P checklist.

## Abbreviations

MMAT: Mixed Methods Appraisal Tool
OECD: Organization for Economic Cooperation and Development
PRISMA-P: Preferred Reporting Items for Systematic Reviews and Meta-Analyses Protocols
RMMA: racial microaggression and macroaggression
UQO: Université du Québec en Outaouais

## References

[1] McAuliffe M, Triandafyllidou A (2022). État de la migration dans le monde 2022 [Report in French]. https://publications.iom.int/system/files/pdf/WMR-2022-FR.pdf.

[2] (2024). International migration outlook 2024. https://www.oecd.org/content/dam/oecd/en/publications/reports/2024/11/international-migration-outlook-2024_c6f3e803/50b0353e-en.pdf.

[3] (2023). Profil du recensement, recensement de la population de 2021 [Article in French]. Statistics Canada.

[4] Morin S (2007). Internationalisation de l’éducation supérieure et formation à distance: le pouvoir d’influence des états occidentaux [Report in French]. https://studylibfr.com/doc/2229944/internationalisation-de-l-%C3%A9ducation-sup%C3%A9rieure-et-formati.

[5] Immigration, Refugees and Citizenship Canada (2024). Temporary residents: study permit holders – monthly IRCC updates - Canada - study permit holders by country of citizenship and year in which permit(s) became effective. Government of Canada.

[6] Bouchard N, Haeck N, Plante M (2021). Le Vivre-Ensemble [Book in French].

[7] Kanouté F, Charette J (2018). La Diversité Ethnoculturelle Dans Le Contexte Scolaire Québécois: Pratiquer Le Vivre-Ensemble [Book in French].

[8] Leroux G (2018). Le défi pluraliste. Éduquer au vivre-ensemble dans un contexte de diversité [Article in French]. Éducation et francophonie.

[9] Potvin M (2014). Diversité ethnique et éducation inclusive: fondements et perspectives [Article in French]. Éducation et sociétés.

[10] Magnan MO, Collins T, Darchinian F, Kamanzi PC, Valade V (2024). Student voices on social relations of race in Québec universities. Race Ethn Educ.

[11] Cotter A (2022). Expériences de discrimination chez les Noirs et les Autochtones au Canada, 2019 [Report in French]. https://www150.statcan.gc.ca/n1/fr/pub/85-002-x/2022001/article/00002-fra.pdf?st=dKrjrWxs.

[12] Sue DW (2017). Microaggressions and “evidence”. Perspect Psychol Sci.

[13] Wang Y, Huang Q, Kim D (2026). Systematic review and meta-analysis: racial-ethnic discrimination and young people's mental health in intensive longitudinal studies. J Am Acad Child Adolesc Psychiatry.

[14] Venkataraman S, Nguyen M, Chaudhry SI (2024). Racial and ethnic discrimination and medical students’ identity formation. JAMA Netw Open.

[15] Luhanga F, Maposa S, Puplampu V, Abudu E (2023). “Let’s call a spade a spade. My barrier is being a Black student”: challenges for Black undergraduate nursing students in a Western Canadian province. Can J Nurs Res.

[16] Pusey-Reid E, Gona CM, Lussier-Duynstee P, Gall G (2022). Microaggressions: Black students’ experiences—a qualitative study. J Prof Nurs.

[17] Lui F, Espinosa A, Anglin DM (2022). Latent class analysis of racial microaggressions and institution-specific racial discrimination at a U.S. minority-serving university: implications for mental health and coping. Am J Orthopsychiatry.

[18] Commission des libérations conditionnelles du Canada (CLCC) / Parole Board of Canada (PBC) (2022). Vers la diversité, l'équité et l'inclusion: rapport du Groupe de travail sur la diversité et le racisme systémique [Report in French]. https://www.canada.ca/content/dam/pbc-clcc/documents/publications/Rapport%20du%20CLCC%20sur%20la%20%20diversit%C3%A9%20et%20le%20racisme%20syst%C3%A9mique.pdf.

[19] Goodwin AKB, Chen GL, Long ACJ (2021). Mental health, well-being, and help-seeking in schools among Black adolescents: the role of discrimination in high-achieving academic settings. Psychol Sch.

[20] Cénat JM, Manoni-Millar S, David A (2025). Racism in education among Black youth in Canada and its association with depression, anxiety, stress, and post-traumatic stress disorder. Res Child Adolesc Psychopathol.

[21] Williams DR, Mohammed SA (2013). Racism and health I: pathways and scientific evidence. Am Behav Sci.

[22] Erving CL, Zajdel R, McKinnon II (2023). Gendered racial microaggressions and Black women's sleep health. Soc Psychol Q.

[23] Rastogi R, Woolverton GA, Lee RM (2024). Microaggression and discrimination exposure on young adult anxiety, depression, and sleep. J Affect Disord.

[24] Berger M, Sarnyai Z (2015). “More than skin deep”: stress neurobiology and mental health consequences of racial discrimination. Stress.

[25] Brody GH, Yu T, Miller GE, Chen E (2015). Discrimination, racial identity, and cytokine levels among African-American adolescents. J Adolesc Health.

[26] Hill LK, Kobayashi I, Hughes JW (2007). Perceived racism and ambulatory blood pressure in African American college students. J Black Psychol.

[27] Heads AM, Glover AM, Castillo LG, Blozis S, Kim SY, Ali S (2021). Perceived discrimination and risk behaviors in African American students: the potential moderating roles of emotion regulation and ethnic socialization. J Racial Ethn Health Disparities.

[28] Boynton MH, O’Hara RE, Covault J, Scott D, Tennen H (2014). A mediational model of racial discrimination and alcohol-related problems among African American college students. J Stud Alcohol Drugs.

[29] (2018). Plan d’action des trois organismes pour l’EDI [Article in French]. Conseil de recherches en sciences naturelles et en génie du Canada (CRSNG).

[30] Okiki C, Giusmin G, Hunter L (2023). “Only for the white.” A qualitative exploration of the lived experiences of Black, Asian and Minority Ethnic midwifery students. Nurse Educ Today.

[31] Pryce-Miller M, Bliss E, Airey A, Garvey A, Pennington CR (2023). The lived experiences of racial bias for Black, Asian and minority ethnic students in practice: a hermeneutic phenomenological study. Nurse Educ Pract.

[32] Langlois S, Gaudreault D (2019). Représentations sociales de la pauvreté et des inégalités au Québec [Article in French]. Rech sociographiques.

[33] Dhume F, Cognet M (2020). Racisme et discriminations raciales à l’école et à l’université: où en est la recherche? [Article in French]. Le Fr Aujourd’hui.

[34] Faye R (2025). Room for discomfort when teaching about racism. Front Educ.

[35] Fanon F (1995). Peau Noire, Masques Blancs [Book in French].

[36] Césaire A (2001). Discourse on Colonialism.

[37] Moher D, Shamseer L, Clarke M (2015). Preferred reporting items for systematic review and meta-analysis protocols (PRISMA-P) 2015 statement. Syst Rev.

[38] Whittemore R, Knafl K (2005). The integrative review: updated methodology. J Adv Nurs.

[39] Furuya-Kanamori L, Lin L, Kostoulas P, Clark J, Xu C (2023). Limits in the search date for rapid reviews of diagnostic test accuracy studies. Res Synth Methods.

[40] Hong QN, Fàbregues S, Bartlett G (2018). Mixed methods appraisal tool (MMAT), version 2018: user guide. http://mixedmethodsappraisaltoolpublic.pbworks.com/w/file/fetch/127916259/MMAT_2018_criteria-manual_2018-08-01_ENG.pdf.

[41] Osei-Kwasi HA, Nicolaou M, Powell K (2016). Systematic mapping review of the factors influencing dietary behaviour in ethnic minority groups living in Europe: a DEDIPAC study. Int J Behav Nutr Phys Act.

[42] Kristoffersson E, Rönnqvist H, Andersson J, Bengs C, Hamberg K (2021). “It was as if I wasn’t there”—experiences of everyday racism in a Swedish medical school. Soc Sci Med.

[43] Ramamurthy A, Bhanbhro S, Bruce F, Collier-Sewell F (2023). Racialised experiences of Black and Brown nurses and midwives in UK health education: a qualitative study. Nurse Educ Today.

[44] (2022). Strengthening primary health care to tackle racial discrimination, promote intercultural services and reduce health inequities: research brief. https://iris.who.int/server/api/core/bitstreams/269925fa-95d1-413c-96b7-61ab2ae8f667/content.

