# Supplementary material for: Manifestations and Impacts of Racial Aggressions on Black Individuals’ Health and Well-Being in University Settings Across Organization for Economic Cooperation and Development Countries: Protocol for an Integrative Review
Source: JMIR Res Protoc. 2026 Sep 25;15:e92748. doi: 10.2196/92748 (PMC13614510; doi:10.2196/92748)
Supplement: Supplementary file 1 [file resprot-v15-e92748-s003.pdf]

N	Name of authors	Year of publication	Title	Country

N	Abstract	Objective	Study design	Method (qualitative, quantitative, mixed method)	Population (country of origin, age)	Main findings in relation to the manifestations of racism (micro and macro) in an academic setting	How is racism perceived by university students?

N	How is racism perceived by university professors?	How is racism perceived by university aids (professors' assistants)?	Examples of microaggressions	Examples of macroaggressions.

N	Main findings in relation to strategies of coping against racism in universities	Health and well-being impacts.	Main findings in relation to protection mechanisms of racial minorities in universities.	Main findings in relation to strategies, rules and regulations applied by universities to fight discrimination and racial aggressions.

N	Recommendations	Limits	Comment
