# Supplementary material for: Manifestations and Impacts of Racial Aggressions on Black Individuals’ Health and Well-Being in University Settings Across Organization for Economic Cooperation and Development Countries: Protocol for an Integrative Review
Source: JMIR Res Protoc. 2026 Sep 25;15:e92748. doi: 10.2196/92748 (PMC13614510; doi:10.2196/92748)
Supplement: Supplementary file 2 [file resprot-v15-e92748-s001.docx]

**S1 Appendix-** **OECD countries and capitals**

| **Countries*** | **Capitals*** |
| --- | --- |
| Australia | Canberra |
| Austria | Vienna |
| Belgium | Brussels |
| Canada | Ottawa |
| Colombia | Bogotá |
| Chile | Santiago |
| Costa Rica | San José |
| Czechia | Prague |
| Denmark | Copenhagen |
| Estonia | Tallinn |
| Finland | Helsinki |
| France | Paris |
| Germany | Berlin |
| Greece | Athens |
| Hungary | Budapest |
| Iceland | Reykjavík |
| Ireland | Dublin |
| Israel | Jerusalem* |
| Italy | Rome |
| Japan | Tokyo |
| Korea (South Korea) | Seoul |
| Latvia | Riga |
| Lithuania | Vilnius |
| Luxembourg | Luxembourg City |
| Mexico | Mexico City |
| Netherlands | Amsterdam (official), The Hague (government) |
| New Zealand | Wellington |
| Norway | Oslo |
| Portugal | Lisbon |
| Slovak Republic (Slovakia) | Bratislava |
| Poland | Warsaw |
| Slovenia | Ljubljana |
| Spain | Madrid |
| Sweden | Stockholm |
| Switzerland | Bern |
| Türkiye (Turkey) | Ankara |
| United Kingdom | London |
| United States | Washington, D.C. |

*The list of OECD member countries is supported by the official OECD source. Capitals are provided from general, widely‑established geographic knowledge.
