# Supplementary material for: Manifestations and Impacts of Racial Aggressions on Black Individuals’ Health and Well-Being in University Settings Across Organization for Economic Cooperation and Development Countries: Protocol for an Integrative Review
Source: JMIR Res Protoc. 2026 Sep 25;15:e92748. doi: 10.2196/92748 (PMC13614510; doi:10.2196/92748)
Supplement: Supplementary file 3 [file resprot-v15-e92748-s002.docx]

# **PubMed/MEDLINE**

| **Number** | **Queries in PubMed/MEDLINE** |
| --- | --- |
| 1 | ("Racism"[Mesh] OR "Prejudice"[Mesh] OR "Social Discrimination"[Mesh] OR "Ethnic and Racial Minorities"[Mesh]  OR racism[tiab] OR racist[tiab] OR "racial bias"[tiab] OR "racial prejudice"[tiab] OR "racial discrimination"[tiab] OR "ethnic discrimination"[tiab] OR "cultural bias"[tiab] OR "anti-black"[tiab] OR "systemic racism"[tiab] OR "structural racism"[tiab] OR "racial inequity"[tiab] OR "racial inequality"[tiab] OR racialized[tiab] OR microaggression*[tiab] OR "race-based"[tiab]) |
| 2 | ("Education, Higher"[tiab] OR "Universities"[Mesh] OR "Schools, Nursing"[Mesh] OR "Schools, Medical"[Mesh] OR "Schools, Dental"[Mesh] OR university*[tiab] OR université*[tiab] OR universidad*[tiab] OR universita*[tiab] OR universität*[tiab] OR universidade*[tiab] OR college*[tiab] OR campus*[tiab] OR "higher education"[tiab] OR "postsecondary education"[tiab] OR "tertiary education"[tiab] OR "vocational school"[tiab] OR polytechnic[tiab] OR institute*[tiab] OR "technical college"[tiab] OR faculty[tiab]) |
| 3 | ("Minority Groups"[Majr] OR "Ethnic Groups"[tiab] OR "African Continental Ancestry Group"[tiab] OR "Ethnic and Racial Minorities"[Majr] OR "racialized" [tiab] OR "racialised" [tiab] OR "racialized people"[tiab] OR "racialized person*"[tiab] OR "racialized group*"[tiab] OR "racialized communit*"[tiab] OR "racial minorities"[tiab] OR "ethnic minorities"[tiab] OR "minority group*"[tiab] OR "minority population*"[tiab] OR "people of color"[tiab] OR "persons of color"[tiab] OR "communities of color"[tiab] OR "people of colour"[tiab] OR "persons of colour"[tiab] OR "communities of colour"[tiab] OR "visible minority"[tiab] OR "visible minorities"[tiab] OR "racial and ethnic group*"[tiab] OR "Black people"[tiab] OR "African descent"[tiab] OR "African American"[tiab] OR "Afro-descendant*"[tiab] OR "BIPOC"[tiab] OR "IBPOC"[tiab] OR "POC"[tiab] OR "minorité visible"[tiab]) |
| 4 | ("OECD countries"[tiab] OR Australia[Mesh] OR Austria[Mesh] OR Belgium[Mesh] OR Canada[Mesh] OR Chile[Mesh] OR Colombia[Mesh] OR "Costa Rica"[Mesh] OR "Czech Republic"[Mesh] OR Czechia[tiab] OR Denmark[Mesh] OR Estonia[Mesh] OR Finland[Mesh] OR France[Mesh] OR Germany[Mesh] OR Greece[Mesh] OR Hungary[Mesh] OR Iceland[Mesh] OR Ireland[Mesh] OR Israel[Mesh] OR Italy[Mesh] OR Japan[Mesh] OR Latvia[Mesh] OR Lithuania[Mesh] OR Luxembourg[Mesh] OR Mexico[Mesh] OR Netherlands[Mesh] OR "New Zealand"[Mesh] OR Norway[Mesh] OR Poland[Mesh] OR Portugal[Mesh] OR Slovenia[Mesh] OR Slovakia[Mesh] OR "Republic of Korea"[Mesh] OR "South Korea"[tiab] OR Spain[Mesh] OR Sweden[Mesh] OR Switzerland[Mesh] OR Turkey[Mesh] OR "United Kingdom"[Mesh] OR UK[tiab] OR "United States"[Mesh] OR USA[tiab]) |

# **PsycINFO**

| **Number** | **Queries in PsycINFO** |
| --- | --- |
| 1 | Abstract {Racism} |
| 2 | Abstract {Higher Education} |
| 3 | Any Field {People of Color} |
| 4 | {Developed Countries} |

# **Scopus/Web of Science**

| **Number** | **Queries in Scopus/Web of Science** |
| --- | --- |
| 1 | KEY ("racism" OR "racist" OR "racial bias" OR "racial prejudice" OR "racial discrimination" OR "ethnic discrimination" OR "cultural bias" OR "anti-black" OR "systemic racism" OR "structural racism" OR "racial inequity" OR "racial inequality" OR "racialized" OR "microaggression" OR "race-based") |
| 2 | KEY ("university*" OR "université*" OR "universidad*" OR "universita*" OR "universität*" OR "universidade*" OR "college*" OR "campus*" OR "higher education" OR "postsecondary education" OR "tertiary education" OR "vocational school" OR polytechnic OR institute* OR "technical college" OR "faculty") |
| 3 | KEY ("minority groups" OR "racialized" OR "BIPOC" OR "people of color" OR "racial minorities") |
| 4 | KEY ("OECD" OR " OECD countries" OR "developed countries" OR "Australia" OR "Austria" OR "Belgium" OR "Canada" OR "Chile" OR "Colombia" OR "Costa Rica" OR "Czechia" OR "Czech Republic" OR "Denmark" OR "Estonia" OR "Finland" OR "France" OR "Germany" OR "Greece" OR "Hungary" OR "Iceland" OR "Ireland" OR "Israel" OR "Italy" OR "Japan" OR "Latvia" OR "Lithuania" OR "Luxembourg" OR "Mexico" OR "Netherlands" OR "New Zealand" OR "Norway" OR "Poland" OR "Portugal" OR "Slovenia" OR "Slovakia" OR "South Korea" OR "Spain" OR "Sweden" OR "Switzerland" OR "Turkey" OR "United Kingdom" OR "UK" OR "United States" OR "USA") |

# **EMBASE**

| **Number** | **Queries in Embase** |
| --- | --- |
| 1 | 'Racism' OR 'racial discrimination' OR 'prejudice' |
| 2 | 'Higher education' OR 'university' OR 'college' OR 'medical school' OR 'nursing school' OR 'vocational education' |
| 3 | 'Minority group' OR 'ethnic group' OR 'black person' OR 'african american' OR 'bipoc' OR 'people of color' OR 'racialized' OR 'racialised' |
| 4 | 'OECD' OR 'OECD countries' OR 'developed countries' |
